# In silico study on *Arabidopsis BAG* gene expression in response to environmental stresses

**DOI:** 10.1007/s00709-016-0961-3

**Published:** 2016-03-22

**Authors:** Ganesh M. Nawkar, Punyakishore Maibam, Joung Hun Park, Su Gyeong Woo, Cha Young Kim, Sang Yeol Lee, Chang Ho Kang

**Affiliations:** 1 Division of Applied Life Science and PMBBRC, Gyeongsang National University, 501 Jinju-daero, Jinju, 52828 Republic of Korea; 2 Eco-friendly Bio-Material Research Center, Korea Research Institute of Bioscience and Biotechnology (KRIBB), Jeongeup, 580-185 Republic of Korea

**Keywords:** Bcl-2 athanogene (BAG), Environmental stress, Transcription profiling, In silico data, Stress-responsive elements

## Abstract

BAG (Bcl-2 athanogene) family proteins are conserved in a wide range of eukaryotes, and they have been proposed to play a crucial role in plant programmed cell death (PCD). During the past decade, with the help of advanced bioinformatics tools, seven homologs of *BAG* genes have been identified in the *Arabidopsis* genome; these genes are involved in pathogen attack and abiotic stress conditions. In this study, gene expression of *Arabidopsis BAG* family members under environmental stresses was analyzed using the Botany Array Resource (BAR) expression browser tool and the in silico data were partially confirmed by qRT-PCR analysis for the selected stress- and hormone-treated conditions related to environmental stresses. Particularly, the induction of *AtBAG6* gene in response to heat shock was confirmed by using GUS reporter lines. The loss of the *AtBAG6* gene resulted into impairment in basal thermotolerance of plant and showed enhanced cell death in response to heat stress. To elucidate the regulatory mechanisms of *BAG* genes, we analyzed ∼1-kbp promoter regions for the presence of stress-responsive elements. Our transcription profiling finally revealed that the *Arabidopsis BAG* genes differentially respond to environmental stresses under the control of specifically organized upstream regulatory elements.

**Electronic supplementary material:**

The online version of this article (doi:10.1007/s00709-016-0961-3) contains supplementary material, which is available to authorized users.

## Introduction

All eukaryotic cells possess a genetically controlled self-destruction mechanism known as programmed cell death (PCD), which is essential for normal development and stress response (Dickman and Fluhr 2013; Kimchi 2007; Lam 2004). As in animals, PCD has been characterized in plants during normal growth and development, as well as in response to various types of stress, including abiotic stress conditions such as heat, cold, salt, and UV radiation and biotic stress conditions such as the hypersensitivity response (HR) to pathogens (Nawkar et al. 2013; Williams and Dickman 2008). Plants share a conserved mechanism of PCD with animal systems, which includes proteins like Bax inhibitor-1 (BI-1), *Arabidopsis* IAP-like protein (AtILP), and BAG family proteins (Ishikawa et al. 2011; Kabbage and Dickman 2008; Kim et al. 2011; Takayama and Reed 2001; Yan et al. 2003).

The BAG family proteins are also well conserved across the yeast, animal, and plant kingdoms. The first member of the BAG family to be characterized was a protein from a mouse embryo complementary DNA (cDNA) library that interacted with human Bcl-2 (Takayama et al. 1995). In *Arabidopsis*, seven members of the BAG protein family were identified using advanced bioinformatics tools, such as profile sequence (Pfam) and profile–profile (FFAS) algorithms (Doukhanina et al. 2006). Like the mammalian BAG proteins, members of the *Arabidopsis* BAG family are also characterized by the presence of a 110–130-amino acid-conserved C-terminal BAG domain (BD). The BD contains three α helices of 30–40 amino acids; the second and third helices are important for co-chaperone activity mediated by direct interaction with the ATPase domain of the heat-shock protein 70 (Hsp70) or heat-shock cognate 70 (Hsc70) chaperones (Briknarova et al. 2001; Fang et al. 2013; Sondermann et al. 2001). The *Arabidopsis* BAG1–4 proteins are characterized by N-terminal ubiquitin-like (UBL) domains, whereas AtBAG5–7 have a plant-specific feature, a calmodulin-binding motif near the BD (Doukhanina et al. 2006). Although information about the *Arabidopsis BAG* genes is limited, recent findings suggest that they act as co-chaperones in various processes related to development and environmental stress. Among the seven members of the *Arabidopsis BAG* gene family, *AtBAG4*, *AtBAG6*, and *AtBAG7* have been studied in relation to the plant PCD in response to cold, heat, UV, pathogen attack, and the unfolded protein response (UPR) (Doukhanina et al. 2006; Williams et al. 2010). Similar to the animal BAG proteins, the concentration of plant BAG proteins in the cell relative to the concentration of Hsp70 is critical for optimal chaperone activity: higher concentrations of BAG proteins may inhibit the refolding activity of Hsp70 by altering its ATP hydrolysis cycle (Doukhanina et al. 2006; Gassler et al. 2001; Hohfeld and Jentsch 1997). Under stress conditions, the refolding activities of Hsp70 and Hsc70 are of particular importance for cell survival; hence, it is important to tightly regulate the levels of these proteins.

To understand the regulatory mechanisms of *BAG* genes in *Arabidopsis*, we measured the transcript levels of all *Arabidopsis BAG* genes in multiple tissues in response to abiotic stress, hormonal treatments, and pathogen attack. We performed in silico analysis of these data by pooling information from previous studies obtained from the publicly available microarray databases. We analyzed the *cis*-regulatory elements present in ∼1-kbp upstream regions of all *Arabidopsis BAG* family genes and further correlated the results with those of the in silico expression analysis. To validate some of these results, we performed qRT-PCR analysis under specific hormone treatment and abiotic stress conditions. We confirmed the induction of *AtBAG6* gene under heat stress by histochemical staining and quantitative fluorogenic β-glucuronidase (GUS) reporter assay. Moreover, basal thermotolerance test and electrolyte leakage experiments clearly confirmed the requirement of *AtBAG6* gene in heat stress tolerance in plants. Finally, we discussed the transcript profiling data in the context of previously reported results.

## Materials and methods

### In silico transcript data analysis

To investigate the absolute expression of *Arabidopsis BAG* family genes in response to hormones and abiotic and biotic stress conditions, data were obtained using the publicly available Botany Array Resource (BAR) expression browser tool (http://bar.utoronto.ca/welcome.htm) (Toufighi et al. 2005). To assess the response to various hormone treatments such as 1-aminocyclopropane-1-carboxylic acid (ACC), methyl jasmonate (MeJA), abscisic acid (ABA), and salicylic acid (SA), we used the AtGenExpress Hormone Series database. To examine the *AtBAG* gene expression in response to different pathogens such as *Pseudomonas syringae* pv *tomato* DC3000, *P. syringae* pv *phasiolicola*, *P. syringae ES4326/ avrRpt2*, *Phytophthora infestanse*, *Botrytis cinerea*, *Erysiphe orontii*, and bacterial and oomycete-derived elicitors, data were extracted from the AtGenExpress Pathogen Series database. To know the response to stress stimuli such as cold, osmotic, salt, drought, oxidative, UV-B, wounding, and heat stress, data were pulled from AtGenExpress Stress Series database. In case of the expression data from hormones, pathogens and abiotic stress treatments were averaged from at least two replicates and the appropriate log_2_-transformed ratios (treated/ control) were used for analysis. The expression values in response to certain hormone and or in stress response without replication were excluded from the analysis.

### Plant materials and growth conditions

All *Arabidopsis* wild-type (WT) and T-DNA mutants were prepared in Columbia (Col-0) ecotype background. We isolated homozygous T-DNA lines *atbag6* (SALK_047959) and *atbag7* (SALK_058247) from ABRC stocks. We used a T-DNA knockout mutant of *AtHSP101*, *hot1*, from our previous studies (Park et al. 2011). The WT (Col-0) seeds were surface-sterilized and then sown on half-strength Murashige and Skoog (1/2 MS, pH 5.7) plates containing 2 % (*w*/*v*) sucrose and 1 % (*w*/*v*) agar for germination. The plates were kept in the dark at 4 °C for 3 days for stratification and then transferred to a controlled environment chamber under 16-h light/ 8-h dark cycle conditions with 100 μmol m^−2^s^−1^ of white light intensity at 22 °C.

### RNA extraction and reverse transcription

For RNA extraction, plants were grown on 1/2 MS plates for 10 days in the vertical position, and then ∼25 seedlings were transferred to 1/2 MS liquid media containing 2 % (*w*/*v*) sucrose and supplemented separately with 10 μM ACC (Sigma-Aldrich, St. Louis, MO, USA), 10 μM MeJA (Sigma-Aldrich, St. Louis, MO, USA), 10 μM ABA;(Sigma-Aldrich, St. Louis, MO, USA), or 0.5 mM SA (Sigma-Aldrich, St. Louis, MO, USA). Abiotic stresses were applied to 18-day-old seedlings by addition of 250 mM NaCl (high salinity) or 250 mM mannitol (osmotic stress) or by incubation at 4 °C (cold) or 37 °C (heat) for 12 h. Shoot and root portions were harvested separately and frozen in liquid nitrogen, and total RNA was extracted using an RNA extraction kit (Qiagen, Valencia, CA, USA) according to the manufacturer’s instructions. NanoDrop ND-1000 spectrophotometer was used for the RNA concentration and purity. To remove genomic DNA contamination, we treated 1 μg RNA with DNase I, RNase-free, and the first strand of cDNA was synthesized using oligo-(d)T primer and RevertAid M-MuLV Reverse Transcriptase (Thermo Scientific, Rockford, IL, USA) according to the manufacturer’s instructions.

### Real-time qRT-PCR analysis

RT-qPCR analysis was performed with CFX 384 Touch™ Real-Time PCR Detection System (Bio-Rad, Hercules, CA, USA) using TOPreal™ qPCR 2X PreMIX (SYBR Green with high ROX) Kit (Enzyomics, Daejeon, Korea) according to the manufacturer’s protocol. In brief, we set a 10-μL reaction volume containing 1 μL cDNA (diluted 1:10), 1 μL of each forward and reverse primer (10 pmol/μL), and 5 μL SYBR Green mix and remaining RNase-free water to make a volume. The PCR cycle conditions followed were 95 °C for 15 min (1×), 95 °C for 10 s/55 °C for 10 s/72 °C for 30 s (40×), followed by a melting curve step to confirm the specificity of the amplified products. Three biological replicates were performed for each sample, and expression levels were normalized against *Actin2*. All primer sequences are listed in Table S1.

### Analysis of *AtBAG6* promoter fused to GUS reporter gene

The ∼1-kb promoter region of the *AtBAG6* gene (P_AtBAG6_) was amplified from *Arabidopsis* genomic DNA by high-fidelity PCR with the primer set of P_AtBAG6_F-attB1/P_AtBAG6_R-attB2 listed in Table S1 and cloned into Gateway entry vector pDONR221 to produce pDONR-P_AtBAG6_ using BP reaction kit (Invitrogen, Carlsbad, CA, USA), by following the manufacturer’s standard protocol. The P_AtBAG6_ region was released from pDONR-P_AtBAG6_ in two steps: firstly, pDONR-P_AtBAG6_ was linearized by restriction digestion enzyme *Nde*I and blunted by DNA Polymerase I Large (Klenow) Fragment (New England Biolabs Inc., Beverly, MA, USA), and, secondly, digested with *Bam*HI. The 35S promoter region of binary vector pCAMBIA1305.1 (P_35S_:*GUS*) was also removed in two steps: firstly, the vector was linearized by restriction digestion enzyme *Bam*HI and blunted by Klenow Fragment and, secondly, digested with *Bgl*II. The resulting P_AtBAG6_ promoter fragment was fused with the GUS reporter gene into the binary vector to generate P_AtBAG6_:*GUS* construct (Fig. S1). Both P_AtBAG6_:*GUS* and P_35S_:*GUS* vectors were used to develop transgenic *Arabidopsis* lines using *Agrobacterium tumefaciens* strain *GV3101* by the floral-dip method (Clough and Bent 1998). Transformed seeds were selected on the MS agar medium supplemented with antibiotics: 10 μg/mL hygromycin (Duchefa, Haarlem, The Netherlands) and 250 μg/mL cefotaxime (Duchefa, Haarlem, The Netherlands). Two-week-old seedlings were subjected to heat stress at 37 °C for 2 h, and then whole seedlings were used for GUS staining as described previously (Jefferson et al. 1987). In brief, seedlings were immersed in the solution containing 1 mg/mL 5-bromo-4-chloro-3-indolyl-β-d-glucuronide, 200 mM sodium phosphate (pH 7.0), 0.5 mM ferricyanide, 0.5 mM ferrocyanide, and 10 mM DTT and then incubated overnight at 37 °C in the dark. Tissues were cleared using 70 % ethanol, and GUS images were taken using a digital camera. For quantitative GUS activity, fluorogenic analyses were performed on protein extracts from the seedlings grown as described above using 4-methylumbelliferyl-β-d-glucuronide (MUG; Sigma-Aldrich, St. Louis, MO, USA) as a substrate as described previously with small modifications (Chen et al. 2008). Briefly, 50 μg of protein extract was added to solution containing 1 mM MUG, 50 mM sodium phosphate (pH 7.0), 10 mM EDTA, 10 mM DTT, 0.1 % Triton X-100, and 15 % methanol to incubate for 15 min at 37 °C. The reaction was stopped using 0.2 M Na_2_CO_3_, and fluorescence was measured with excitation 364 nm and emission 447 nm on a Gemini XPS Fluorometer (Molecular Devices, Sunnyvale, CA, USA) and data represented as arbitrary fluorescence units per microgram of protein. Assays were conducted using samples from three biologically independent experiments.

### Basal thermotolerance test

For the basal thermotolerance test, seedlings were grown on solid nutrient medium containing 2 % (*w*/*v*) sucrose with similar growth conditions as described in earlier section (Larkindale et al. 2005). Plates containing 5-day-old seedlings were sealed with plastic electric tape and submerged in a water bath at temperature 45 °C for an indicated time, and then plates were removed from the water bath and maintained under the previous normal growth conditions using the same light/dark cycles. Basal thermotolerance was determined using the survival rate.

### Electrolyte leakage measurements

For electrolyte leakage measurement, 7 day-old seedlings were placed in 5 mL of deionized water and placed on a shaker at 22 °C, and first conductivity of the solutions was determined by Orion 3 Star conductometer (Thermo Electron Cooperation, USA) and was designated as reading “A.” The same plants were kept in a hot water bath at 45 °C for 15 min in a dark condition and allowed samples to recover at 22 °C for 3 h on a shaker, and again conductivity of the solutions was measured and was designated as “B.” After autoclaving samples for 15 min, the conductivity was re-measured to obtain the total amount of ions in the cell. The ion leakage was expressed as a percentage of the ratio of the conductivity measured at A and B to that after autoclaving.

### In silico *cis*-regulatory promoter sequence analysis

To find the regulatory *cis*-elements present in the promoter region of the *Arabidopsis BAG* family genes, we used the publicly available Plant *cis*-acting regulatory DNA elements (PLACE; http://www.dna.affrc.go.jp/PLACE/), Plant cis-acting regulatory elements (PlantCARE; http://bioinformatics.psb.ugent.be/webtools/plantcare/html), and Plant promoter database version 3.0 (PPDB; http://ppdb.agr.gifu-u.ac.jp/ppdb/cgi-bin/index.cgi) databases (Hieno et al. 2014; Higo et al. 1999; Lescot et al. 2002). We selected ∼1-kbp regions upstream of the translation initiation codon of individual *Arabidopsis BAG* genes and analyzed them for the presence of stress- or hormone-responsive elements.

### Statistical analysis

All values reported in experiments for qRT-PCR, fluorometric GUS assay, and electrolyte leakage measurements are mean of three replicates. Statistical analyses were performed using Student’s *t* test. *P* values were calculated using GraphPad QuickCalcs (available online at http://www.GraphPad.com/).

## Results

### Expression analysis of *Arabidopsis BAG* genes in response to hormonal perturbations

**Fig. 1:**
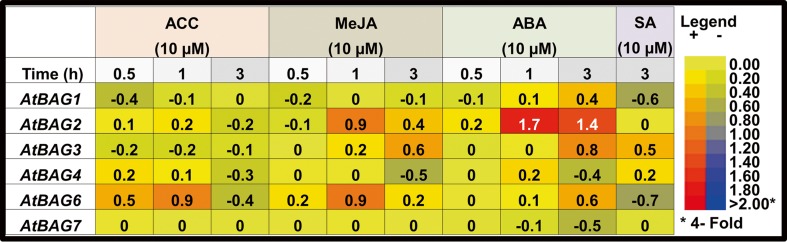
Expression of *Arabidopsis BAG* genes in response to environmental stress-related hormonal treatments. *Values* from the BAR expression browser are represented as log_2_-transformed ratios (treated/control) in the table. The *color scale* is given on the *right. ACC* 1-aminocyclopropane-1-carboxylic acid, *MeJA* methyl jasmonate, *ABA* abscisic acid, *SA* salicylic acid

First, we systematically analyzed the expression profiles of seven *Arabidopsis BAG* genes by electronic Northern blotting using the BAR expression browser (Toufighi et al. 2005). To determine the modulation of *Arabidopsis BAG* gene expression in response to various hormones, we calculated the log_2_-transformed ratios of treated vs. control samples (Fig. 1). The data for *AtBAG5* gene were not available. The expression level of *AtBAG1*, *AtBAG4*, and *AtBAG7* altered to a less extent in response to the hormonal treatments (as most of their values of log_2_-transformed ratios range between −0.5 and +0.5). Expression of *AtBAG2* was induced highly by ABA treatment and slightly by MeJA treatment. Transcript level of *AtBAG3* was slightly upregulated in response to MeJA, ABA, and SA. The expression level of *AtBAG6* transcript was slightly increased by treatment with ACC, MeJA, and ABA, while it was slightly repressed by SA.

### Analysis of *Arabidopsis BAG* gene expression in response to pathogen attack

**Fig. 2:**
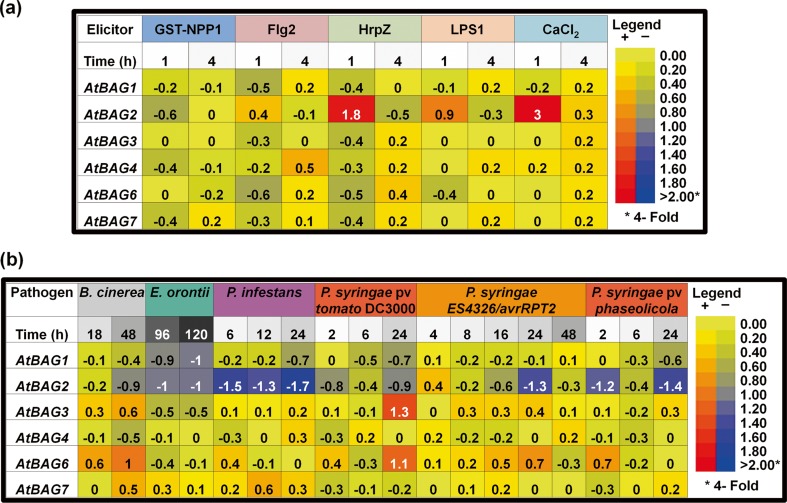
Expression of *Arabidopsis BAG* genes in response to elicitors and pathogens. **a** Response to elicitors [chemical elicitor, CaCl_2_; bacterial-derived elicitors, flagellin 2 (Flg2), hairpin Z (HrpZ), lipopolysaccaride (LPS); fungal elicitors, necrosis-inducing Phytophthora protein 1 (NPP1)]. **b** Response to pathogens [virulent (*Pseudomonas syringae* pv *tomato* DC3000), avirulent (*P. syringae ES4326/avrRpt2*), and non-host (*P. syringae* pv *phaseolicola*) bacteria; biotrophic (*Erysiphe orontii*), hemi-biotrophic (*Phytophthora infestans*), and necrotrophic (*Botrytis cinerea*) fungi]. *Values* are represented as log_2_-transformed ratios (treated/control). The *color scale* is given on the *right*

Next, we investigated *Arabidopsis BAG* gene expression in response to elicitors (Fig. 2a) and pathogens (Fig. 2b). Interestingly, expression of *AtBAG2* was induced rapidly in response to chemical elicitor CaCl_2_ and bacterial-derived elicitors such as hairpin Z (HrpZ) and LPS lipopolysaccaride (LPS) but down-regulated in response to oomycete-derived elicitors such as necrosis-inducing Phytophthora protein 1 (NPP1). Furthermore, *AtBAG2* expression was highly down-regulated in response to non-host and avirulent *Pseudomonas syringae* and *Pseudomonas infestans* and moderately down-regulated in response to *Erysiphe orontii*, *Botrytis cinerea*, and virulent *P. syringae*. Although *AtBAG1* showed similar responses such as *AtBAG2* to various pathogens, the level of down-regulation of *AtBAG1* gene was lower than that of *AtBAG2*. The expression level of *AtBAG3* was slightly higher in response to virulent *P. syringae* than *B. cinera. AtBAG6* transcript was accumulated moderately in response to *P. syringae* pv *tomato* DC3000 and *B. cinera* and weakly in response to *P. syringae ES4326/avrRpt2*. We did not observe a huge alteration in the expression of *AtBAG4* and *AtBAG7* after pathogen challenge (Fig. 2b).

### Expression profile of *Arabidopsis BAG* genes in response to abiotic stresses

**Fig. 3:**
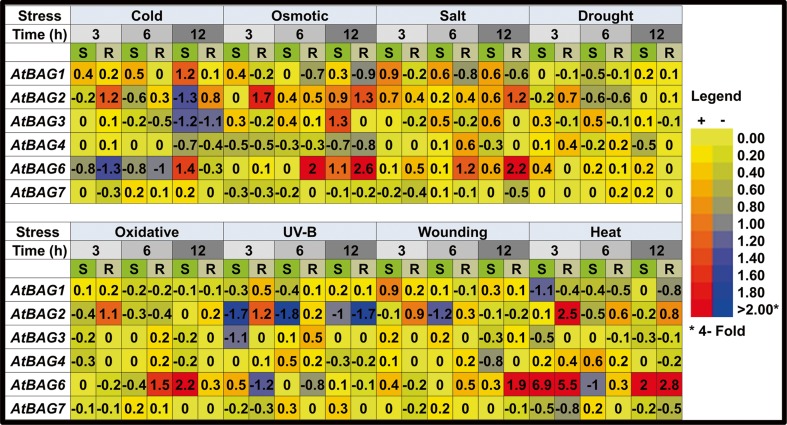
Expression of *Arabidopsis BAG* genes in response to abiotic stresses. *Values* are represented as log_2_-transformed ratios (treated/control). The *color scale* is given on the *right. S* shoot tissue, *R* root tissue

Next, we monitored the transcriptional response of *Arabidopsis BAG* genes under various abiotic stress conditions such as cold, osmotic, salt, drought, oxidative, UV-B, wounding, and heat stress (Fig. 3). Expression of *AtBAG1* was slightly induced in shoots in response to cold, salt, and wounding, but it was repressed under heat stress. *AtBAG2* was moderately to highly induced in roots in response to drought, cold, salt, oxidative, osmotic, and heat stress. In response to UV-B, the expression of *AtBAG2*, *AtBAG3* (in shoots), and *AtBAG6* (in roots) was moderately down-regulated. *AtBAG3* exhibited mild induction in response to salt and drought, and moderately high induction under osmotic stress (at late time points), but slight repression under heat and osmotic stress (at early time points). Expression of *AtBAG4* was slightly repressed by cold, osmotic stress, and wounding. Expression of *AtBAG7* exhibited weak modulation in response to various stresses, whereas *AtBAG6* was strongly induced in response to heat, oxidative, osmotic, salt, and wounding stress.

### Validation of *Arabidopsis BAG* gene expression by qRT-PCR

**Fig. 4:**
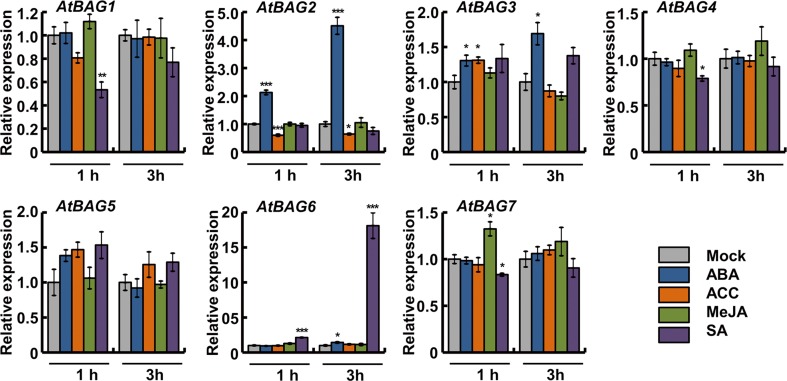
Determination of relative transcript abundances of *Arabidopsis BAG* genes under different hormone treatments by qRT-PCR. Ten-day-old seedlings were transferred to liquid 1/2 MS media supplemented with 10 μM abscisic acid (ABA), 10 μM 1-aminocyclopropane-1-carboxylic acid (ACC), 10 μM methyl jasmonate (MeJA), and 0.5 mM salicylic acid (SA). Samples were harvested at 1 and 3 h for total RNA isolation. Expression of *AtBAG* genes was determined by qRT-PCR. Three biological replicates were averaged; *error bars* indicate standard error of the mean. *Asterisks* indicate statistically significant differences between normal and phytohormone treatments as calculated using the Student *t* test (*P < 0.05, **P < 0.01, and ***P < 0.001, respectively)

**Fig. 5:**
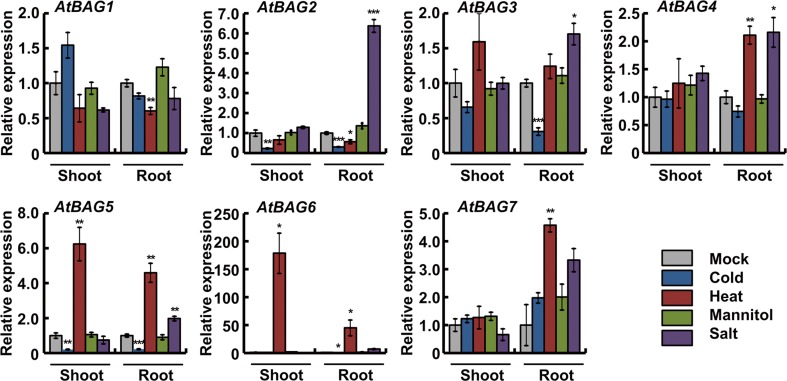
Determination of relative transcript abundances of *Arabidopsis BAG* genes under different stress conditions by qRT-PCR. Eighteen-day-old seedlings were transferred to 1/2 MS liquid media and incubated at 4 °C (cold) or 37 °C (heat) or treated with 250 mM mannitol (osmotic stress) or 250 mM NaCl (high salinity) for 12 h. Shoot and root samples were harvested for total RNA isolation. Expression of *AtBAG* genes was determined by qRT-PCR. Three biological replicates were averaged; *error bars* indicate standard error of the mean. *Asterisks* indicate statistically significant differences between normal and abiotic stress treatments as calculated using the Student *t* test (*P < 0.05, **P < 0.01, and ***P < 0.001, respectively)

After systematic in silico analysis of *Arabidopsis BAG* gene expression under various stress conditions and hormone treatments, we validated some of these in silico data by performing qRT-PCR. To determine whether our hormone or stress conditions induced the desired effect, we analyzed the expression of relevant marker genes: *PDF1.2* for MeJA, *PR1* for SA, *RD29B* for ABA, *COR15a* for cold, *HSFA6a* for salt and osmotic stress, *HSP101* for heat, and *RD29A* for salt and cold (Figs. S1 and S2). Based on the induction patterns of these marker genes, relevance of the stress treatments was confirmed. Moreover, we observed a high degree of correlation between the in silico and qRT-PCR data for the *Arabidopsis BAG* genes (Figs. 4 and 5). Our qRT-PCR data revealed that *AtBAG1* expression was slightly induced in shoot under cold, and repressed by heat and salt stress treatment and slightly repressed by SA. Expression of *AtBAG2* was induced in response to ABA and salt treatments in root but suppressed by cold and heat stress, which is consistent with the in silico data (Fig. 3). In response to ABA and SA, *AtBAG3* expression was slightly induced while it was down-regulated by cold. Expression of *AtBAG4* showed minor changes in response to different hormonal treatments. However, we observed *AtBAG4* gene induction by salt and heat treatment and reduction by cold in the root tissues. Our qRT-PCR results provide important insights into *AtBAG5* expression under different environmental constraints because the data were not available in the BAR expression browser. Expression of *AtBAG5* was induced in both shoot and root under heat stress condition, but showed few changes in response to hormonal treatments, for example, slightly induced in response to ABA, ACC, and SA. Consistent with the in silico data, we observed greater induction of *AtBAG6* specifically in root by salt stress, and it also induces in response to heat stress in both shoot and root. Expression of *AtBAG6* gene was elevated by SA treatment. Expression of *AtBAG7* was slightly upregulated in response to MeJA and also abiotic stress treatments such as heat and salt stress in root tissue.

### Validation of *AtBAG6* gene expression under heat stress by GUS reporter assay

**Fig. 6:**
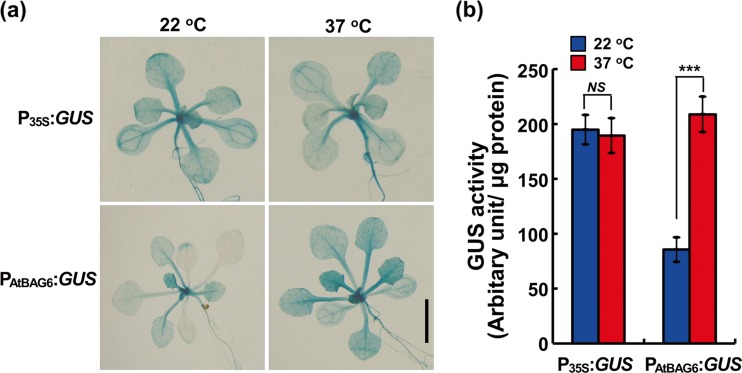
Visualization of P_AtBAG6_:*GUS* activity. **a** Histochemical analysis of P_35S_:*GUS* and P_AtBAG6_:*GUS* lines under a normal growth condition (22 °C) and after heat stress (37 °C). At least three independent transgenic plants were used for each analysis. *Bar* = 5 mm. **b** Quantitative fluorogenic GUS activity analysis of P_35S_:*GUS* and P_AtBAG6_:*GUS* lines under a normal growth condition (22 °C) and after heat stress (37 °C). Three biological replicates were averaged; *error bars* indicate standard deviation (S.D.) *Asterisks* indicate statistically significant differences between normal and heat stress conditions as calculated using the Student *t* test (***P < 0.001 and *NS*, no significance)

Our in silico expression analysis of *AtBAG* genes in response to different abiotic stresses clearly indicated the highest expression levels of *AtBAG6* under heat stress condition. These results were also consistent with our qRT-PCR results. Thus, to validate the same results, we adopted the GUS reporter assay system. We constructed plasmids for GUS reporter experiments and investigated the GUS activity driven by the *AtBAG6* promoter (P_AtBAG6_) and cauliflower mosaic virus (CaMV) 35S promoter (P_35S_) (Fig. S3). Under normal condition (22 °C), we observed strong GUS expression in P_35S_:*GUS* lines in all tissues of 5-day-old seedlings while P_AtBAG6_:*GUS* lines showed weaker GUS expression mostly limited to hypocotyls and trace amounts in roots and leaves (data not shown). For heat stress treatment, we grow plants for 2 weeks at normal condition (22 °C) which were then subjected to heat stress at 37 °C for 2 h. We found a significant increase in GUS activity under heat stress condition (37 °C) in P_AtBAG6_:*GUS* lines, but there were no visible changes in P_35S_:*GUS* lines (Fig. 6a). To quantify GUS activity, we conducted fluorogenic GUS activity assays on protein samples from the same seedlings described above. The fluorogenic analysis results for P_35S_:*GUS* lines indicated higher constitutive GUS activity compared to P_AtBAG6_:*GUS* lines at 22 °C temperature (Fig. 6b). On the other hand, at 37 °C for 2 h, heat shock results in a significant increase in GUS activity only in P_AtBAG6_:*GUS* lines; P_35S_:GUS lines did not exhibit differences (Fig. 6b). Taken together, our results strengthen in silico analysis as well as qRT-PCR data obtained from the *AtBAG6* gene expression under heat stress conditions.

### Physiological role of *AtBAG6* in basal thermotolerance

**Fig. 7:**
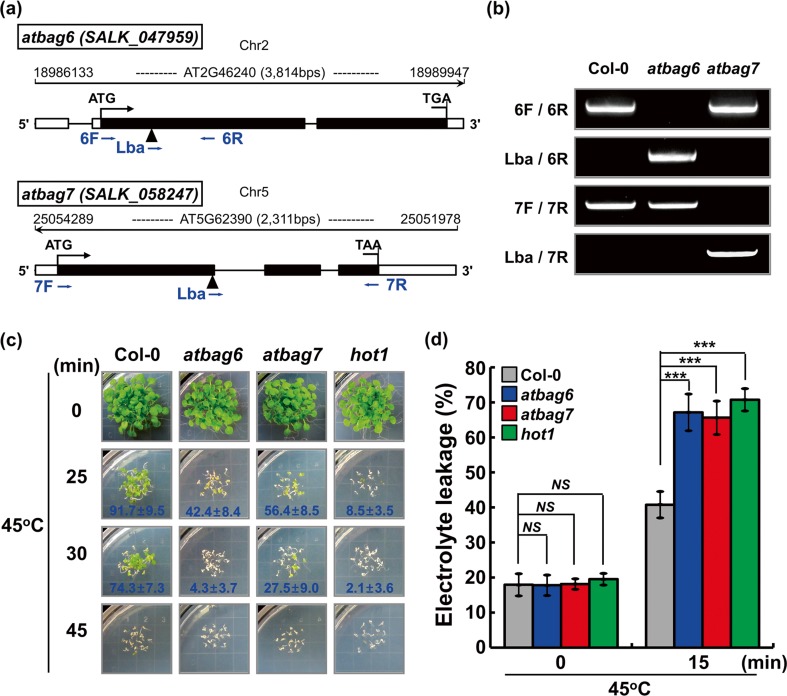
Basal thermotoleranc response of Col-0, *atbag6*, *atbag7*, and *hot1.* **a** Schematic diagrams of predicted gene structure of *ATBAG6* (*upper panel*) and *ATBAG7* (*lower panel*). *Black arrow* represents start codon ATG (exons, *filled boxes*; introns, a *horizontal black line*; 5′ and 3′ UTRs, *blank boxes* at both ends). The positions of the T-DNA insertion are indicated by *black arrow heads. Blue arrows* represent the primer positions used for genotyping. **b** The T-DNA insertion lines *atbag6* and *atbag7* were genotyped using the primer combinations shown as in **a. c** Basal thermotolerance test conducted using Col-0, *atbag6*, *atbag7*, and *hot1* line by growing plants under a normal growth condition (22 °C) for 5 days, subjected to heat shock (45 °C) for the indicated time, and then allowed to recover for more than 1 week, and photographs were taken. Survival rate (percent; mean ± SD) of the heat-treated plants for 25 and 30 min was obtained from three independent experiments and shown at the *bottom* of the image. **d** Electrolyte leakage was measured using 7-day-old plants grown at normal growth condition (22 °C) by incubating in 5 mL deionized water, subjected to heat shock (45 °C) for 15 min, and allowed to recover 3 h, and again electrolyte leakage was measured. Total ion was measured after autoclaving the samples, and ion leakage was expressed as a percentage of the ratio of the conductivity measured at 22 and 45 °C to that after autoclaving. Data represents a standard deviation (SD) calculated from three biological replicates. *Asterisks* indicate statistically significant differences between different genotypes under normal and heat stress conditions as calculated using the Student *t* test (***P < 0.001 and *NS*, no significance)

Our qPCR data and GUS data clearly confirmed the induction of *AtBAG6* gene expression in response to heat shock. We further investigated to know the role of *AtBAG6* in heat stress response. To test basal thermotolerance, we isolated and characterized an *A. thaliana* homozygous *atbag6* mutant line (SALK_047959) carrying a T-DNA insertion in the first exon of *AtBAG6* and the *atbag7* mutant line (SALK_058247) carrying a T-DNA insertion in the first exon region of *AtBAG7* (Fig. 7a) which was confirmed by genomic DNA PCR (Fig. 7b). As a positive control of heat sensitivity assay, we used a T-DNA knockout mutant of *A. thaliana HSP101*, *hot1*, which is required for both basal and acquired thermotolerance (Hong and Vierling 2000; Hong and Vierling 2001; Queitsch et al. 2000). When grown under normal conditions, no obvious phenotypic differences were observed among WT, *atbag6*, *atbag7*, and *hot1*, but when 5-day-old seedlings were exposed to 45 °C for the indicated time interval and then recovered at 22 °C, it showed that *atbag6, atbag7*, and *hot1* failed to recover growth as compared to WT (Fig. 7c). To strengthen thermotolerance results, we conducted an electrolyte leakage assay which is a good indicator of damaged plasma membrane of plants in response to heat stress (Hong et al. 2003). In response to heat shock (45 °C for 15 min), all genotypes including WT, *atbag6*, *atbag7*, and *hot1* showed a significant increase in percent ion leakage, but *atbag6*, *atbag7*, and *hot1* showed a significantly higher level as compared to WT (Fig. 7d). These results suggest that the loss of *AtBAG6* enhances the plants’ sensitivity to heat stress.

### Regulatory element analysis in promoter of *Arabidopsis BAG* gene

**Fig. 8:**
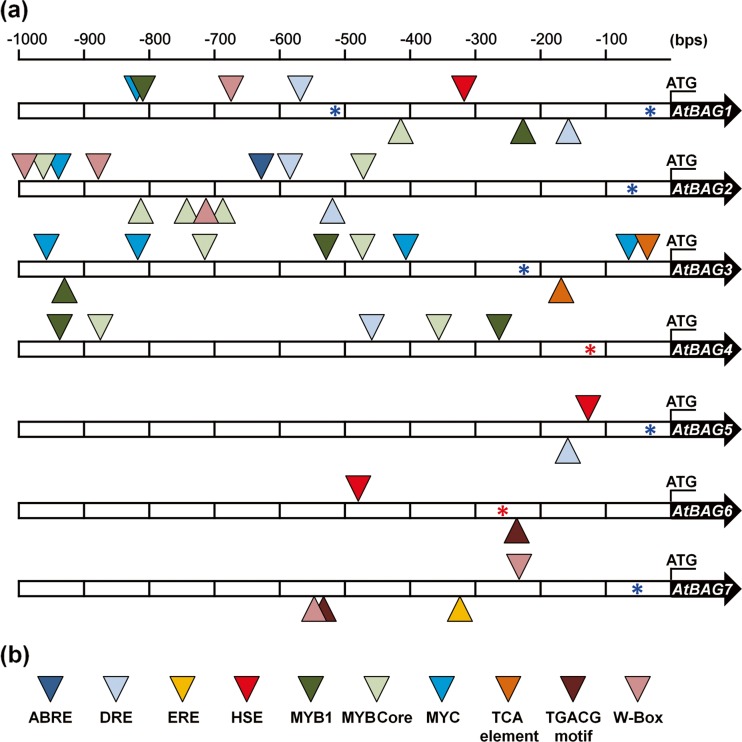
Various *cis*-elements present in the ∼1-kbp promoter regions of *Arabidopsis BAG* genes. **a** The promoter analyses for *AtBAG1*∼*7* were performed using the PLACE, PlantCare, and PPDB databases. *Colored arrowheads* indicate the relative positions of the different elements. *Asterisks* (*) indicate the TSS (*red ones* from PPDB database and *blue ones* from literature (Doukhanina et al. 2006; Kabbage and Dickman 2008). The translational initiation codons (ATG) are indicated. Ruler for the promoter sequences is shown above the schemes. **b** *Colored arrowheads* used in **a** to indicate various *cis*-elements present in the ∼1-kbp promoter regions of the *Arabidopsis BAG* genes

Gene expression in response to various stimuli is largely regulated by conserved *cis*-elements present in the promoter region. Hence, we analyzed ∼1-kbp promoter regions of *Arabidopsis BAG* genes, using the publicly available PLACE, PlantCARE, and PPDB databases (Hieno et al. 2014; Higo et al. 1999; Lescot et al. 2002). In the promoter regions of *Arabidopsis BAG* genes, we observed multiple stress-related *cis*-elements, including the MYC consensus sequence, the MYB-binding site, the drought-responsive element (DRE), the heat-shock element (HSE), and hormone-responsive elements such as ABA-responsive element (ABRE), ethylene-responsive element (ERE), TGACG motif, TCA element, and W-box (Fig. 8 and Table 1). In order to assess the relevant existence of all these regulatory elements in the promoter region of *AtBAG* genes, we considered their respective positions as compared to the transcription start site (TSS) using the PPDB database. Since the TSS information for *AtBAG4*, *AtBAG5*, and *AtBAG6* was not available, we searched the related literatures and found additional TSS information for *AtBAG4* and *AtBAG6* promoters (Doukhanina et al. 2006; Kabbage and Dickman 2008). Taken together, we integrated all data from the BAR expression confirmed by qRT-PCR to the promoter analysis and summarized information in Table 1 to impart biological meanings for the presence of regulatory *cis*-elements and specific motifs in the promoters of *Arabidopsis BAG* genes.

**Table 1:** Summary report for BAR, qRT-PCR data, and promoter motif analysis

Genes	BAR data confirmed by qRT PCR	Motif	Sequence^a^	Position from ATG	Reference
*AtBAG1*	Slightly induced in shoot in response to cold, but slightly repressed in both shoot and root in response to heat and salt stresses	DRE MYC MYB1 MYB core HSE	CCGAC CANNTG WAACCA CNGTTR AAAACTTTC	−166, −576 −826 ^−^224, −812 −407, −1022 −307	–
*AtBAG2*	Induced in response to ABA Induced in root in response to salt, but repressed in both shoot and root in response to cold and heat	ABRE DRE MYC MYB Core	TACGGTC CCGAC CANNTG CNGTTR	−627 −520, −582 −942, −1058 −479, −698, −742, −806, −960	–
*AtBAG3*	Slightly induced in response to ABA and SA. Induced in response to salt, but repressed in both shoot and root in response to cold	TCA element MYC MYB core MYB 1	GAGAAGAATA CANNTG CNGTTR WAACCA	−34, −169 −69, −402, −812, −962 −492, −701, −1011 −524, −933	–
*AtBAG4*	Repressed in root in response to cold, but induced in salt and heat in root	DRE MYB core MYB1 MYC	CCGAC CNGTTR WAACCA CANNTG	−467 −366, −888 −266, −935 −145, −147, −156, −276	(Doukhanina et al. 2006; Kabbage and Dickman 2008)
*AtBAG5*	Induced in both shoot and root in response to heat Repressed in response to cold	DRE HSE	CCGAC AAAAACTTTC	−154 −137	–
*AtBAG6*	Highly induced in response to SA and slightly by MeJA Induced in both shoot and root response to heat	TGACG motif HSE	TGACG AGAAAATTCC	−221 −488	(Doukhanina et al. 2006; Kang et al. 2006)
*AtBAG7*	Slightly induced in ACC and MeJA	ERE TGACG motif	ATTTCAAA TGACG	−317 −524	–

*DRE* drought-responsive element, *HSE* heat-shock element, *ABRE* ABA-responsive element, *ERE* ethylene-responsive element

^a^IUPAC nucleotide code: W = A/T, N = A/T/G/C, R = A/G

## Discussion

The BAG family is a multifunctional group of proteins that act as co-chaperones that regulate cell signaling, growth, and development and are involved in environmental stress responses (Doukhanina et al. 2006; Kim et al. 2011). Thus, it is important to know their expression in response to different biotic and abiotic stresses and regulatory mechanisms. We systematically analyzed the expression levels of *Arabidopsis BAG* genes in response to different hormones and biotic and abiotic stresses using the in silico approach. It is well known that plant hormones play important roles not only in growth and development but also in diverse biotic and abiotic stress responses. Thus, in this study, stress-related phytohormones such as ABA, ethylene, MeJA, and SA were targeted. Our in silico analysis showed that ABA induces expression of *AtBAG3* and *AtBAG6* at moderate levels and *AtBAG2* at higher levels, which was also confirmed by qRT-PCR. Furthermore, the expression of *AtBAG2* and *AtBAG3* was induced in response to salt stress and repressed by cold treatment. These results are in accordance with previous studies which proposed that the expressions of *Arabidopsis BAG* genes are regulated by ABA, which is important for adaptive responses to various environmental stresses (Doukhanina et al. 2006; Kang et al. 2006). Moreover, we confirmed the presence of conserved stress-responsive *cis*-elements, such as DRE, ABRE, MYC, HSE, and MYB-binding sites, in the promoter regions of *Arabidopsis BAG* genes (Fig. 8 and Table 1); these elements may be important for the regulation of their expression. The presence of conserved stress-responsive motifs in the promoter regions of *Arabidopsis BAG* genes, and the induction of these genes in response to ABA, suggests that they play a role in adaptive responses to different environmental stresses, such as cold, drought, and high salinity (Table 1). The relevance of the presence of conserved motifs in the promoters of *Arabidopsis BAG* genes to stress responses, as well as the identities of the regulators of gene expression, could be addressed in future studies.

The key roles for ethylene, MeJA, and SA have been documented in regulating plant defense responses against different pathogens. In particular, ethylene and MeJA are usually involved in defense against necrotrophic pathogens and insect pests while SA activates defense responses against biotrophic and hemi-biotrophic pathogens and also induces systemic acquired resistance (SAR) (Govrin and Levine 2002). Our qRT-PCR data showed that the expression of *AtBAG6* is increased in response to SA and MeJA, suggesting its role in innate immunity and basal defense response. In addition to this, our in silico analysis showed the slight induction of *AtBAG6* in response to pathogens *B. cinerea* and *P. syringae*. These results are also supported by previous reports which suggest that *AtBAG6* plays a role in the host defense mechanism and is upregulated in response to SA and that T-DNA insertion mutants of *AtBAG6* (*atbag6*) have an elevated susceptibility to the necrotrophic fungal pathogen *B. cinerea* (Doukhanina et al. 2006; Kang et al. 2006). Recently, it has also been reported that the activation of *AtBAG6* by aspartyl protease cleavage is an important event to trigger autophagy and plant defense against fungal pathogen (Li et al. 2016). The classical example of plant PCD is the HR, observed during plant–microbe interactions (Lam et al. 2001). Moreover, it has been suggested that the overexpression of the AtBAG6 cell death domain (CDD) results in an HR-like phenotype in plant leaves, resulting in PCD (Kang et al. 2006). Taken together, these data confirm the regulatory role of *AtBAG6* in plant PCD and imply that the function of *AtBAG6*-induced cell death is not simply to kill individual cells, but rather to save the whole plant.

We observed that *AtBAG2* expression was specifically downregulated in response to different pathogens, but induced by bacterial-derived elicitors, suggesting its role in plant defense system against pathogen attack. These results are also supported by the presence of four W-box motifs in the promoter region of *AtBAG2*; the W-box is a known WRKY-binding site and acts as a negative regulatory element for the inducible expression of *AtWRKY18* (Chen and Chen 2002). These observations suggest the possibility that the *AtBAG2* level in response to pathogen attack may be tightly regulated by the W-box motif, in a manner similar to regulation of *AtWRKY18*. In this regard, future studies could investigate the up- or down-regulation of *AtBAG2* in response to the pathogen-induced HR pathway.

In plants, not only biotic stresses but also abiotic stresses such as cold, drought, salt, heat, and UV induce the PCD and pose serious constraints on plant growth and yield (Nawkar et al. 2013; Williams and Dickman 2008). Previous studies described the upregulation of *AtBAG4* under cold stress and also showed that constitutive expression of *AtBAG4* in transgenic tobacco imparts tolerance to cold, salt, UV, and oxidative stress (Doukhanina et al. 2006; Kabbage and Dickman 2008). Our in silico and qRT-PCR data also describe that *AtBAG4* was induced by salt and heat in root tissue, but we could not observe increased expression of *AtBAG4* in response to cold. This discrepancy with previous reports might be due to a difference in the growth conditions as well as cold treatment conditions. We followed conditions mentioned at BAR and gave cold stress treatment to plants by transferring plants to 4 °C for 12 h, while in previous reports, they used −20 °C for 10 min.

In the present study, our systematic analysis revealed that *AtBAG6* was clearly up-regulated in response to heat stress which was further supported by qRT-PCR and quantitative fluorogenic GUS assay results (Figs. 5 and 6). Thus, we pursue to check the role of *AtBAG6* in basal thermotolerance of plants and used *AtBAG7* mutant as a positive control. Although ER stress induced by cold, heat, or the chemical agent tunicamycin does not affect the transcript level of ER-localized *AtBAG7*, this protein still plays an important role in delaying stress-induced PCD by directly interacting with a molecular chaperone, BIP2 (Williams et al. 2010). The transcriptional response of *AtBAG7* supports the idea that the co-chaperone activity of *AtBAG7* is essential for delaying PCD induced by ER stress, as hypothesized in earlier studies (Williams et al. 2010). Moreover, there is a possibility that *AtBAG7* may be regulated at the protein level: for example, a recent study suggested that ER-localized *AtBAG7* translocates to the nucleus in response to heat stress and regulates the UPR pathway (Williams et al. 2014). In this report, we found that *atbag6* plants similar to the *atbag7* line were impaired in basal thermotolerance. Moreover, these mutants showed increased electrolyte leakage in response to heat stress indicating more plasma membrane damage. The heat stress sensitive phenotype of *atbag6* may not be attributed to defect in autophagy because heat stress-induced autophagy is not regulated by *AtBAG6* (Li et al. 2016). There may be a possibility that, similar to *AtBAG7* co-chaperone activity, *AtBAG6* is also involved in heat stress tolerance. This report demonstrates the importance of *BAG* genes for plant survival under stress conditions. Apart from ER membrane, organelles such as chloroplast and mitochondria are also playing an important role in regulating PCD in plants (Nawkar et al. 2013; Williams and Dickman 2008). The localization of *AtBAG5* has been predicted to be mitochondria and may be an important component in regulating mitochondria-regulated cell death mechanism. We observe the presence of HSE in the promoter of *AtBAG5* which was induced in response to heat. Thus, it would be interesting to check the role of *AtBAG5* in heat stress tolerance.

In conclusion, our study not only confirms previous reports but also suggests new hypotheses regarding the roles of *BAG* genes in the regulation of PCD induced by various biotic and abiotic stresses in plants. For example, we have described in detail the conserved motifs present in the promoter regions of *Arabidopsis BAG* genes, which are involved in their regulation by upstream transcription factors; future studies could attempt to elucidate the molecular mechanisms underlying this regulation. In addition, it will be interesting to investigate the regulatory networks involving *Arabidopsis BAG* family genes under multiple environmental conditions. The accumulated data suggest that *Arabidopsis* BAG proteins are involved in plant defenses against biotic or abiotic stresses, whether these involve induction or inhibition of cell death; however, the individual functions and precise mechanisms of the BAG proteins could be studied in more detail in the future. In that context, we hope that our retrieval of data related to the expression of *Arabidopsis BAG* genes provides a useful preliminary introduction and motivates related research.

## Electronic supplementary material

Below is the link to the electronic supplementary material.

Fig. S1 (PDF 120 kb)

Fig. S2 (PDF 149 kb)

Fig. S3 (PDF 146 kb)

Table S1 (PDF 38 kb)
