# Supplementary material for: In silico study on Arabidopsis BAG gene expression in response to environmental stresses
Source: Protoplasma. 2016 Mar 22;254(1):409–21. doi: 10.1007/s00709-016-0961-3 (PMC5216074; doi:10.1007/s00709-016-0961-3)
Supplement: Supplementary file 1 — (PDF 120 kb) [file 709_2016_961_MOESM1_ESM.pdf]

SUPPLEMENTARY MATERIALS:

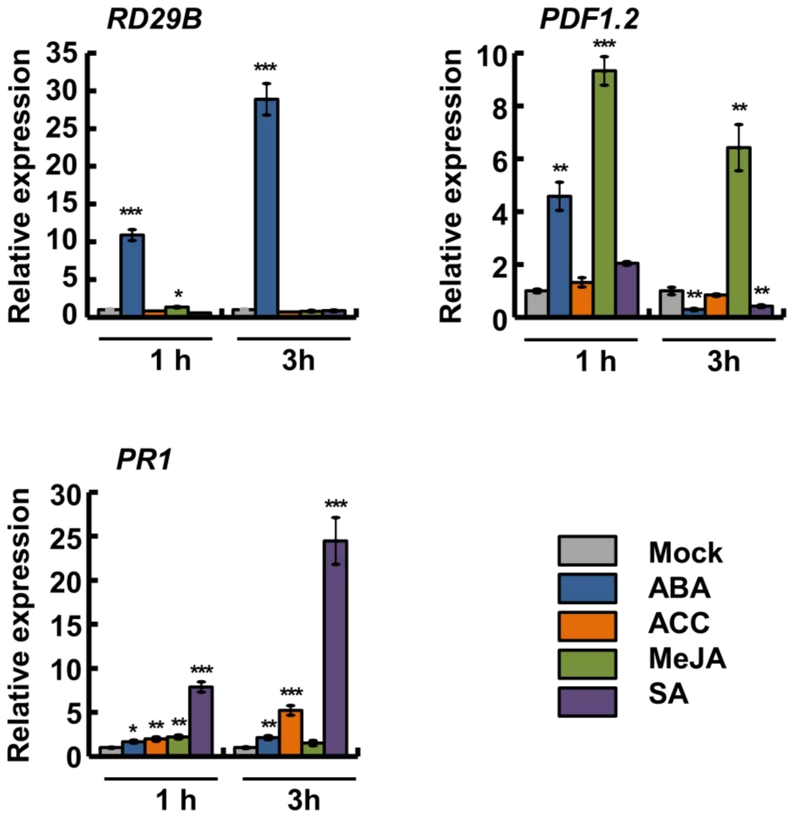

Fig. S1 Determination of relative transcript abundances of marker genes under different hormone treatments by qRT-PCR. 10-days-old seedlings were transferred to liquid 1/2 MS media supplemented with 10 µM abscisic acid (ABA), 10 µM 1-aminocyclopropane-1-carboxylic acid (ACC), 10 µM methyl jasmonate (MeJA) and 0.5 mM salicylic acid (SA). Samples were harvested at 1h and 3h for total RNA isolation. Expression of marker genes was determined by qRT-PCR. Three biological replicates were averaged; error bars indicate standard error of the mean. Asterisks indicates statistically significant differences between normal and phytohormones treatments as calculated using the Student's t test (*$P < 0.05$, ** $P < 0.01$, and ***$P < 0.001$, respectively). *RD29B*, *responsive to desiccation 29B* (AT5G52300); *PDF1.2*, *plant defensing 1.2* (AT5G44420); *PR1*, *pathogenesis-related gene 1* (AT2G14610).
