# Supplementary material for: In silico study on Arabidopsis BAG gene expression in response to environmental stresses
Source: Protoplasma. 2016 Mar 22;254(1):409–21. doi: 10.1007/s00709-016-0961-3 (PMC5216074; doi:10.1007/s00709-016-0961-3)
Supplement: Supplementary file 2 — (PDF 149 kb) [file 709_2016_961_MOESM2_ESM.pdf]

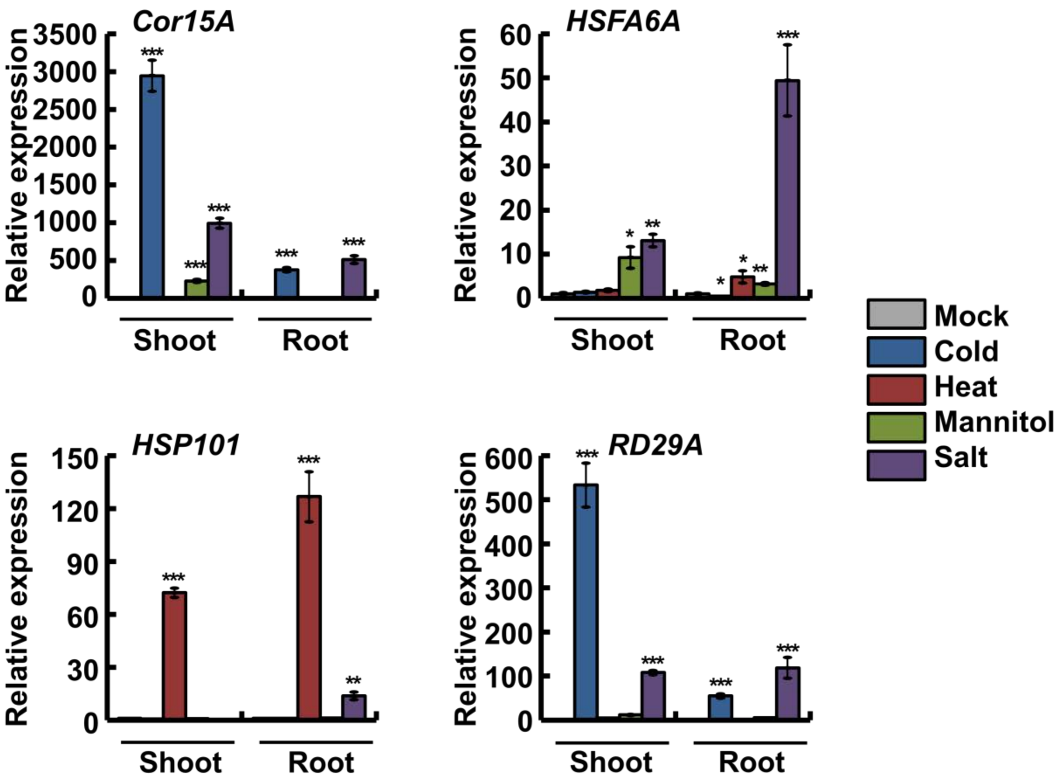

Fig. S2 Determination of relative transcript abundances of marker genes under different stress conditions by qRT-PCR. 18-days-old seedlings were transferred to 1/2 MS liquid media and incubated at 4°C (cold) or 37°C (heat) or treated with 250 mM mannitol (osmotic stress) or 250 mM NaCl (high salinity) for 12 hours. Shoot and root samples were harvested for total RNA isolation. Expression of marker genes was determined by qRT-PCR. Three biological replicates were averaged; error bars indicate standard error of the mean. Asterisks indicates statistically significant differences between normal and abiotic stress tratments as calculated using the Student's t test (*$P < 0.05$, ** $P < 0.01$, and ***$P < 0.001$, respectively). *COR15A*, *cold-regulated 15A* (AT2G42540); *HSFA6A*, *heat shock transcription factor A6A* (AT5G43840); *HSP101*, *heat shock protein 101* (AT1G74310); *RD29A*, *responsive to desiccation 29A* (AT5G52310).
