# Supplementary material for: In silico study on Arabidopsis BAG gene expression in response to environmental stresses
Source: Protoplasma. 2016 Mar 22;254(1):409–21. doi: 10.1007/s00709-016-0961-3 (PMC5216074; doi:10.1007/s00709-016-0961-3)
Supplement: Supplementary file 3 — (PDF 146 kb) [file 709_2016_961_MOESM3_ESM.pdf]

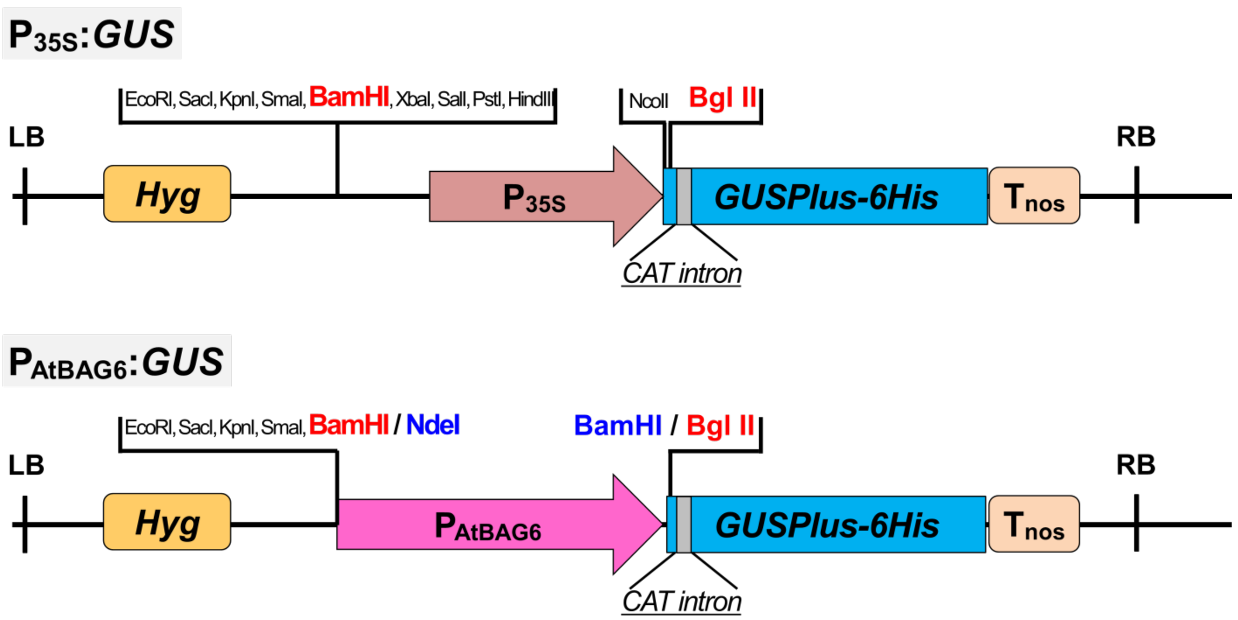

Fig. S3 Schematic diagrams of the P_{35S}:*GUS* and P_{AtBAG6}:*GUS* constructs. RB, right border; LB, left border; P_{35S}, Cauliflower mosaic virus (*CaMV*) *35S* promoter; P_{AtBAG6}, *AtBAG6* gene promoter; *Hyg*, hygromycin phosphotransferase coding region; *GUSPlus-6His*, β-glucuronidase gene fused to six histidine codons; *CAT intron*, catalase gene intron; T_{nos}, nopaline synthase terminator. The insertion position of the P_{AtBAG6} in the vector is indicated by the restriction enzyme sites, *Bam*HI/*Nde*I and *Bam*HI/*Bgl*II. - See more in the Materials and Methods part.
