# Supplementary material for: In silico study on Arabidopsis BAG gene expression in response to environmental stresses
Source: Protoplasma. 2016 Mar 22;254(1):409–21. doi: 10.1007/s00709-016-0961-3 (PMC5216074; doi:10.1007/s00709-016-0961-3)
Supplement: Supplementary file 4 — (PDF 38 kb) [file 709_2016_961_MOESM4_ESM.pdf]

Table S1 Sequences of primers used in this research.

Gene	Locus	Primer	Sequences (5’-3’)	Purpose
Actin2	AT3G18780	Actin2-F	TGATGCACTTGTGTGTGACAA	qRT-PCR
		Actin2-R	GGGACTAAAACGCAAAACGA	
AtBAG1	AT5G52060	AtBAG1-F	AGATGATGAGAAACAAACCGACAA	
		AtBAG1-R	CGGAACCTCTACCGCCATTA	
AtBAG2	AT5G62100	AtBAG2-F	AAGGCAACCGCAAACGAA	
		AtBAG2-R	TGTCAGCATTTCCCGTTCCT	
AtBAG3	AT5G07220	AtBAG3-F	GTCTCGACCTGGAGGAATGG	
		AtBAG3-R	ACGTGGCACATCGGAGTTC	
AtBAG4	AT3G51780	AtBAG4-F	AGCTGTAAGCACTGAGTGGGAAT	
		AtBAG3-R	TGGCGGAGGAGGGTTCA	
AtBAG5	AT1G12060	AtBAG5-F	TCCCGATTGAAACTCCATTACC	
		AtBAG5-R	GCGGTGGCGTTTGTTTTG	
AtBAG6	AT2G46240	AtBAG6-F	CCTGGCAACGGATTCTAAGC	
		AtBAG6-R	GGAATTGTTGTCGAGGAAGC	
AtBAG7	AT5G62390	AtBAG7-F	GTTCGAGGAGCGAAGAGATCA	
		AtBAG7-R	GCGGGTCTACCACATCTAACATT	
COR15A	AT2G42540	COR15A-F	AACGAGGCCACAAAGAAAGC	
		COR15A-R	CAGCTTCTTTACCCAATGTATCTG	
PDF1.2	AT5G44420	PDF1.2-F	GCTTCCATCACCCTTATCTTC	
		PDF1.2-R	GCCGGTGCGTCGAAA	
HSP101	AT1G74310	HSP101-F	CACAATCTCCTCCACCTGATGA	
		HSP101-R	GCACGACGAATGACCTTAATAAGA	
PR1	AT2G14610	PR1-F	GTCTCCGCCGTGAACATGT	
		PR1-R	CGTGTTCGCAGCGTAGTTGT	
HSFA6A	AT5G43840	HSFA6A-F	CCCTAGATGCTTTAAGCACAATAATTTCT	
		HSFA6A-R	ATCGATGTCATCACCATACTCTTCTGG	
RD29A	AT5G52310	RD29A-F	GTTACTGATCCCACCAAAGAA	
		RD29A-R	GGAGACTCATCAGTCACTTCCA	
RD29B	AT5G52300	RD29B-F	GCAAGCAGAAGAACCAATCA	
		RD29B-R	CTTTGGATGCTCCCTTCTCA	
AtBAG6	AT2G46240	P_{AtBAG6}F-attB1[*]	aaaaagcaggcatatgAACGAAACCCAAATAATT	Cloning
		P_{AtBAG6}R-attB2[*]	agaaagctgggTTGTGATGGATCCATGTA	
AtBAG6	AT2G46240	6F	ATGATGCCTGTGTACATGGAT	genomic PCR
		6R	AGAGAAGGTACTTCGTCTGTCTCC	
AtBAG7	AT5G62390	7F	ATGACTTTGTTCCATAGACTCGAT	
		7R	TTCTTCTTCCGTTTCAAGCAT	
T-DNA		LBa	TGGTTCACGTAGTGGGCCATCG	

* attB1 and attB2 sites are underlined.
